# Artificial intelligence assisted cytological detection for early esophageal squamous epithelial lesions by using low‐grade squamous intraepithelial lesion as diagnostic threshold

**DOI:** 10.1002/cam4.4984

**Published:** 2022-06-29

**Authors:** Bin Yao, Yadong Feng, Kai Zhao, Yan Liang, Peilin Huang, Juncai Zang, Jie Song, Mengjie Li, Xiaofen Wang, Huazhong Shu, Ruihua Shi

**Affiliations:** ^1^ The Laboratory of Image Science and Technology Southeast University Nanjing China; ^2^ Froeasy Technology Development CO., LTD Red Maple Park of Technological Industry Nanjing China; ^3^ Department of Gastroenterology, School of Medicine Zhongda Hospital Southeast University Nanjing China; ^4^ Department of Gastroenterology Jintan First People's Hospital Affiliated to Jiangsu University Changzhou China; ^5^ School of Medicine Zhongda Hospital Southeast University Nanjing China

**Keywords:** AI‐assisted diagnosis, cytology, early esophageal squamous cell cancer, precursor lesion, screening

## Abstract

**Background:**

Manual cytological diagnosis for early esophageal squamous cell carcinoma (early ESCC) and high‐grade intraepithelial neoplasia (HGIN) is unsatisfactory. Herein, we have introduced an artificial intelligence (AI)‐assisted cytological diagnosis for such lesions.

**Methods:**

Low‐grade squamous intraepithelial lesion or worse was set as the diagnostic threshold for AI‐assisted diagnosis. The performance of AI‐assisted diagnosis was evaluated and compared to that of manual diagnosis. Feasibility in large‐scale screening was also assessed.

**Results:**

AI‐assisted diagnosis for abnormal cells was superior to manual reading by presenting a higher efficiency for each slide (50.9 ± 0.8 s vs 236.8 ± 3.9 s, *p* = 1.52 × 10^−76^) and a better interobserver agreement (93.27% [95% CI, 92.76%–93.74%] vs 65.29% [95% CI, 64.35%–66.22%], *p* = 1.03 × 10^−84^). AI‐assisted detection showed a higher diagnostic accuracy (96.89% [92.38%–98.57%] vs 72.54% [65.85%–78.35%], *p* = 1.42 × 10^−14^), sensitivity (99.35% [95.92%–99.97%] vs 68.39% [60.36%–75.48%], *p* = 7.11 × 10^−15^), and negative predictive value (NPV) (97.06% [82.95%–99.85%] vs 40.96% [30.46%–52.31%], *p* = 1.42 × 10^−14^). Specificity and positive predictive value (PPV) were not significantly differed. AI‐assisted diagnosis demonstrated a smaller proportion of participants of interest (3.73%, [79/2117] vs.12.84% [272/2117], *p* = 1.59 × 10^−58^), a higher consistence between cytology and endoscopy (40.51% [32/79] vs. 12.13% [33/272], *p* = 1.54 × 10^−^8), specificity (97.74% [96.98%–98.32%] vs 88.52% [87.05%–89.84%], *p* = 3.19 × 10^−58^), and PPV (40.51% [29.79%–52.15%] vs 12.13% [8.61%–16.75%], *p* = 1.54 × 10^−8^) in community‐based screening. Sensitivity and NPV were not significantly differed. AI‐assisted diagnosis as primary screening significantly reduced average cost for detecting positive cases.

**Conclusion:**

Our study provides a novel cytological method for detecting and screening early ESCC and HGIN.

## Graphical abstract

This study has provided an automated classfication for esophageal epithelial squmaous cells. This AI‐based system is competent in identifying atypical squamous cells and low‐grade squamous intraepithelial lesion.Hence, our research may contribute to promotion of cytological detection of early esophageal squmaous cell carcinoma.

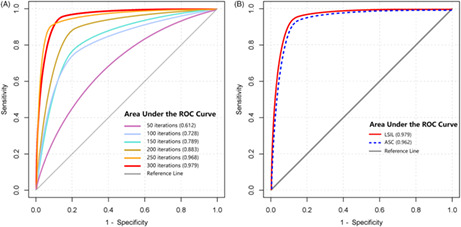

## 1 INTRODUCTION

Esophageal squamous cell carcinoma (ESCC) is one of the leading causes of cancer mortality, and the incidence of ESCC is increasing worldwide.^1^ Due to clinical significance, large‐scale endoscopy screening for early ESCC and high‐grade intraepithelial neoplasia (HGIN) has been conducted in population‐based pipeline for several decades in China.^1^, ^2^ Since large‐scale endoscopy screening is high‐costing, cytological approaches have gained much interest as a pre‐endoscopy screening for detecting such lesions.^3^ However, it is not widely performed due to poor outcomes. And, one obstacle is lack of a novel method for the evaluation of cytological samples.^4^

Manual diagnosis for early ESCC and HGIN is always performed in the way that cytologists manually scan the whole slide. Besides the heavy workload, some crucial problems in assessing all available slides from population‐based screening remain to be unsolved, including follows. Firstly, atypical squamous cell (ASC)‐or‐worse is adopted as the diagnostic threshold for detecting abnormal cells of interest. Due to the low‐rank clinical significance of ASC,^5^ the outcomes were unsatisfactory.^6^ Secondly, although diagnostic criteria for cytological diagnosis for squamous cells have been proposed,^7^ it is difficult to classify such cells by manual evaluation of morphological features only.^5^ Thirdly, since cytological diagnosis for a few abnormal cells is experience‐dependent, it is difficult to achieve high interobserver reproducibility, even among experienced cytopathologists.^8^ Therefore, it is appreciated to develop a method with novel diagnostic efficiency and high accuracy.

Application of artificial intelligence (AI)‐assisted cytological diagnosis has been developed to detect dysplasia cells,^9^ and has made pathological diagnosis convenient.^10^, ^11^ Recently, we have reported our pilot work of AI‐assisted cytological detection for esophageal squamous epithelial lesions.^12^, ^13^ Although outcomes were superior to those by manual diagnosis, some false‐negative cases were detected due to adoption of ASC‐or‐worse the diagnostic threshold, and some unnecessary endoscopy examinations were performed. With further training and validation, this AI‐system is more accurate in classifying esophageal squamous cells, which provides a rigid diagnostic threshold for cytological detection for potential lesions.^11^ Herein, low‐grade squamous intraepithelial lesion (LSIL)‐or‐worse was adopted as the diagnostic threshold for cytological detection for early ESCC and HGIN. The main aim was to assess the performance of AI‐assisted cytological detecting for early ESCC and HGIN by using this setting, along with its probability and feasibility in community‐based screening.

## 2 MATERIALS AND METHODS

### 2.1 Study design

An AI‐based cytological diagnosis system from Froeasy Tech Co., which is designed for automated classification of esophageal squamous epithelial cells, has been applied for cytological detecting early ESCC and HGIN. By setting LSIL‐or‐worse as the diagnostic threshold, the performances of AI‐assisted diagnosis were evaluated in patients with pathologically confirmed esophageal lesions and asymptomatic participants with high risks of ESCC. This study has been registered on chineseclinicaltrials.gov (ChiCTR1900028524). The protocol conformed to the ethical guidelines of the 1975 Declaration of Helsinki, and has been approved by review board of Zhongda Hospital Southeast University, with Ref No. 2019ZDSYLL092‐P01. Written consent was obtained from each participant before enrollment into this study.

### 2.2 Patients

On the basis of our previous study,^13^ an additional patient cohort, including 139, 30, 14, and 34 patients with early ESCC, HGIN and low‐grade intraepithelial neoplasia (LGIN), and reflux erosive esophagitis (REE), respectively, were enrolled from Zhongda Hospital Southeast University and Jintan First People's Hospital Affiliated to Jiangsu University. Between July 2019 and April 2020, sponge‐derived esophageal epithelial samples were successfully retrieved from 164, 61, 41, and 34 patients with early ESCC, HGIN and LGIN, and REE, respectively.

### 2.3 Sample collection, treatment, and cytological diagnosis

**FIGURE 1:**
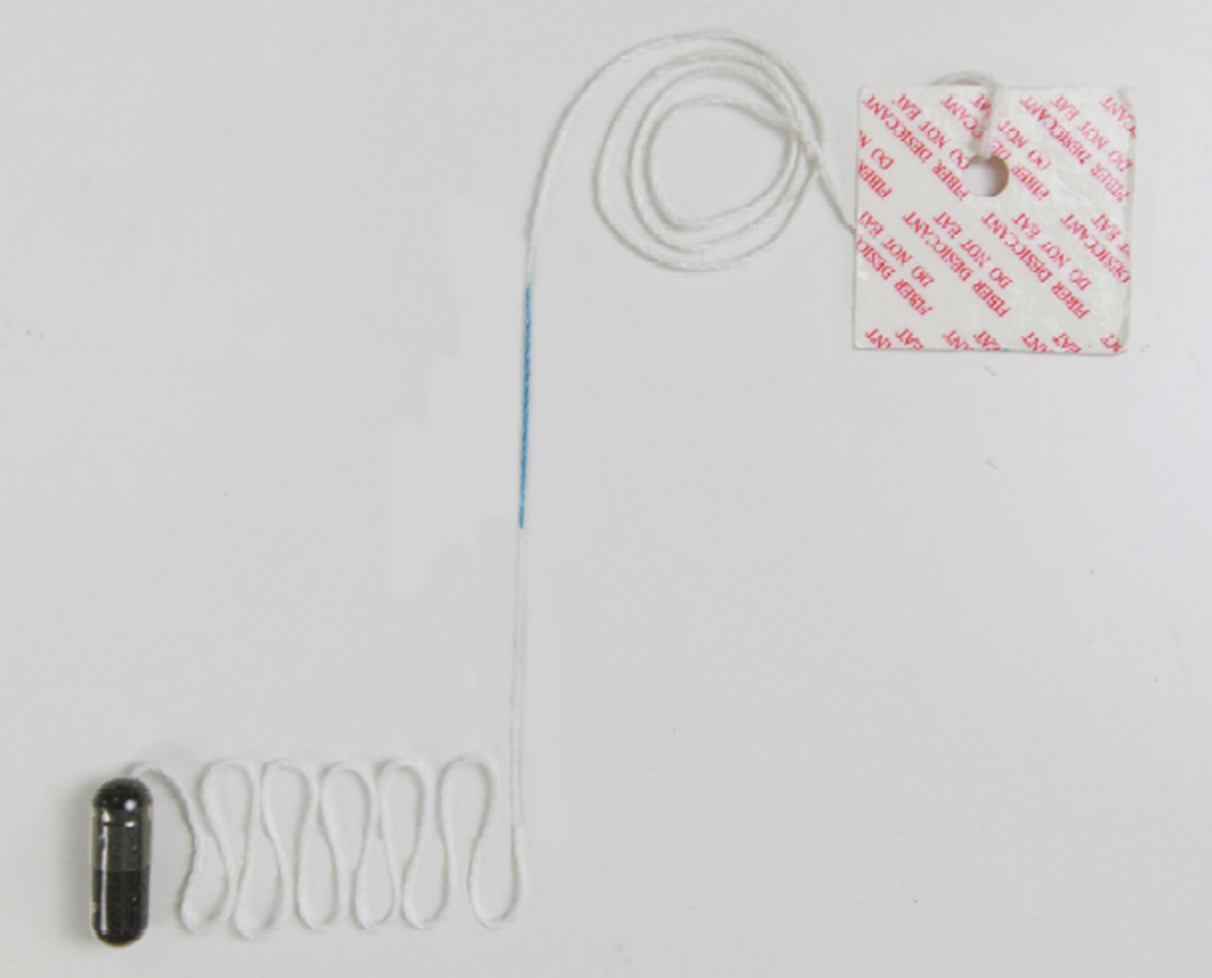
Details of the capsule contained sponge device by Froeasy Tech Co. (Shikang I®/Eso Heal I™). The capsule sponge sampler contains a sponge mesh that was restrained in a capsule and attached string with an index mark on the distal end.

Cytological samples were collected by patients swallowing a sponge device (Shikang I®/Eso Heal I™, Froeasy Tech Co., Chinese invention patent is pending, 201911049863.7, Figure 1) and were stored in cell preservation solution (China invention patent, ZL201810314036.5). Slides were prepared by a liquid‐based thin layer cell preparation technique followed by Feulgen‐Eosin staining (China invention patent, ZL 201710732464.5).

Esophageal squamous cells were classified into five categories,^14^ including non‐intraepithelial malignancy (NILM), ASC, LSIL, high‐grade squamous intraepithelial lesion (HSIL), and squamous cell carcinoma (SCC).

### 2.4 Brief introduction of AI‐assisted diagnosis system

The AI‐assisted diagnosis system, which is available online at http://pms.n‐ecdc.com/, was developed by supervised deep learning. The architecture of this model was based on convolutional neural networks, including Res Net and Seg Net. Taking advantages of Feulgen‐Eosin staining, morphological and nuclear characteristics are available. As we previously reported,^12^, ^13^ digital images of cells, with 89 × 89 dpi each cell, along with their nuclei, were obtained by an automated scan by a digital scanner equipped with a double charge‐coupled‐device camera. Cells and relevant nuclei were labeled by expert cytopathologists, and were subjected to deep learning. In the initial stage, 55,600 esophageal epithelial cells were used as a training set.^13^ In this study, additional 1,905,600 digital images of esophageal epithelial cells and relevant nuclei were used for further training. Every 100,000 images of NILM, ASC, LSIL, HSIL, and SCC were used for internal validation. And, about every 3,500,000 esophageal squamous cells were used for external validation.

### 2.5 Cytological detection of histopathologically confirmed early ESCC and HGIN

Feulgen‐Eosin stained slides were randomly selected from patients with histopathologically confirmed REE, LGIN, HGIN, and early ESCC, which were not involved in development of AI‐assisted diagnosis system. Three competent pathologists, who were masked to patients' histopathological results and not involved in the establishment of this AI‐assisted diagnosis system, were asked to complete sequentially independent diagnoses by manual cytology and AI‐assisted diagnosis. A manual system from Foreasy Tech Co. (version 2.0), which consists of a microscope with a ×20 objective, a condenser numerical aperture (NA) of 0.75 and a camera adapter of factor 0.87, a double charge‐coupled‐device camera, and a computer with a high‐resolution monitor, was used for manual classification of cells. Manual diagnosis was made based on DNA content and morphological features. AI‐assisted diagnosis was performed as AI‐reading for abnormal cells followed by manual confirmation.

### 2.6 Community‐based screening

From July 2019 to August 2020, 2090 asymptomatic participants (aged ≥40 years) with high risks of ESCC from 10 communities in Nanjing were recruited.^13^ And, additional 27 participants were enrolled between August 2020 and October 2020. Hence, 2117 participants were enrolled for cytological and endoscopic screening of early ESCC and HGIN. There were 1082, 1376, 103, 346, 672, 476, and 766 participants with smoking, heavy drinking, a history of SCC, family history of ESCC, high‐temperature food preference, rapid eating, or multiple (≥2) risk factors, respectively. All participants underwent sponge cytological specimen retrieval and endoscopic examination for detecting early ESCC and HGIN. And cytological samples of esophageal epithelial squamous cells were collected before endoscopy. Lugol's iodine staining was performed in endoscopic examination under sedation, and endoscopic biopsy was performed as necessary. Cytological outcomes were evaluated and compared to those of endoscopic and pathological results.

### 2.7 Statistical analysis

The diagnostic accuracy, sensitivity, specificity, positive predictive value (PPV), and negative predictive value (NPV) were evaluated, and 95% confidence intervals (CIs) were calculated using the Clopper–Pearson method. A receiver operating characteristic (ROC) curve was used to show the diagnostic performance of the deep learning algorithm in classifying esophageal squamous epithelial cells. A Student's *t* test was used for the comparison of continuous variables. All statistical tests were performed using the SPSS statistical software for Windows, version 20.0 (SPSS Inc.) and R software (version 3.5.3).

## 3 RESULTS

### 3.1 Efficiency of the automated diagnosis

**FIGURE 2:**
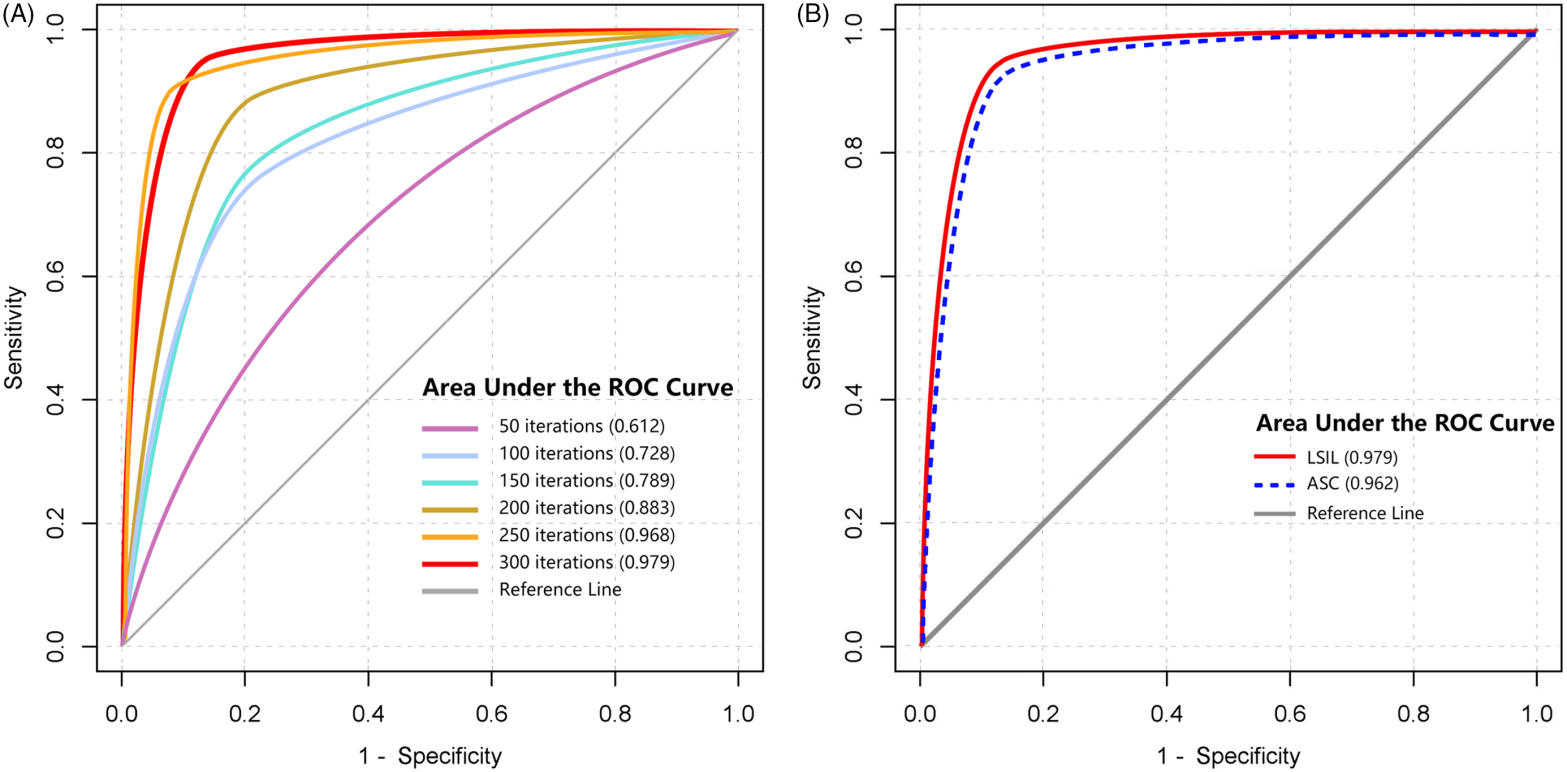
Receiver operating characteristic (ROC) curve of training performances in training and diagnostic performances by trained AI model. (A) A plot of ROCs of per‐interaction performances in training set. As shown in the figure, the area under the ROC curves (AUC) values for 50, 100, 150, 200, 250, and 300 interactions were 0.612, 0.728, 0.789, 0.883, 0.968, and 0.979, respectively. (B) Diagnostic performances of this trained model in identifying ASC and LSIL, respectively. Relevant values of AUC were 0.962 and 0.979, respectively. AI: artificial intelligence.

After 300 iterations through the entire training set, the training procedure was concluded due to no improvement in diagnostic accuracy (Figure 2A). As shown in Figure 2B, the AI model demonstrated novel diagnostic performances in identifying ASC and LSIL. In the internal validation, the consistency between automated classification and expert pathologist's diagnosis was about 99.58%. There were 1032 (1.03%) ASC misdiagnosed as LSIL, and 727 (0.73%) LSIL misdiagnosed as ASC. By using about each 3,500,000 esophageal squamous cells for external validation, the rates of correct classification were 100%, 96.27%, 97.86%, 99.6%, and 100% for NILM, ASC, LSIL, HSIL, and SCC, respectively. About 6300 (0.18%)LSIL were misclassified as ASC, and 42,011 (1.2%) ASC misidentified as LSIL.

### 3.2 Performance in detecting histopathologically confirmed early ESCC and HGIN

Slides from 127, 28, 14 and 24 patients with early ESCC, HGIN, LGIN, and REE, respectively, were selected for manual diagnosis and AI‐assisted diagnosis. The time taken to read each slide by AI‐assisted diagnosis was significantly shorter than that of manual diagnosis (50.9 ± 0.8 s vs 236.8 ± 3.9 s, *p =* 1.52 × 10^−76^). The interobserver agreement of identifying LSIL or worse by AI‐assisted reading was better than that of manual reading (93.27% [95% CI, 92.76%–93.74%] vs. 65.29% [95% CI, 64.35%–66.22%], *p* = 1.03 × 10^−84^).

**FIGURE 3:**
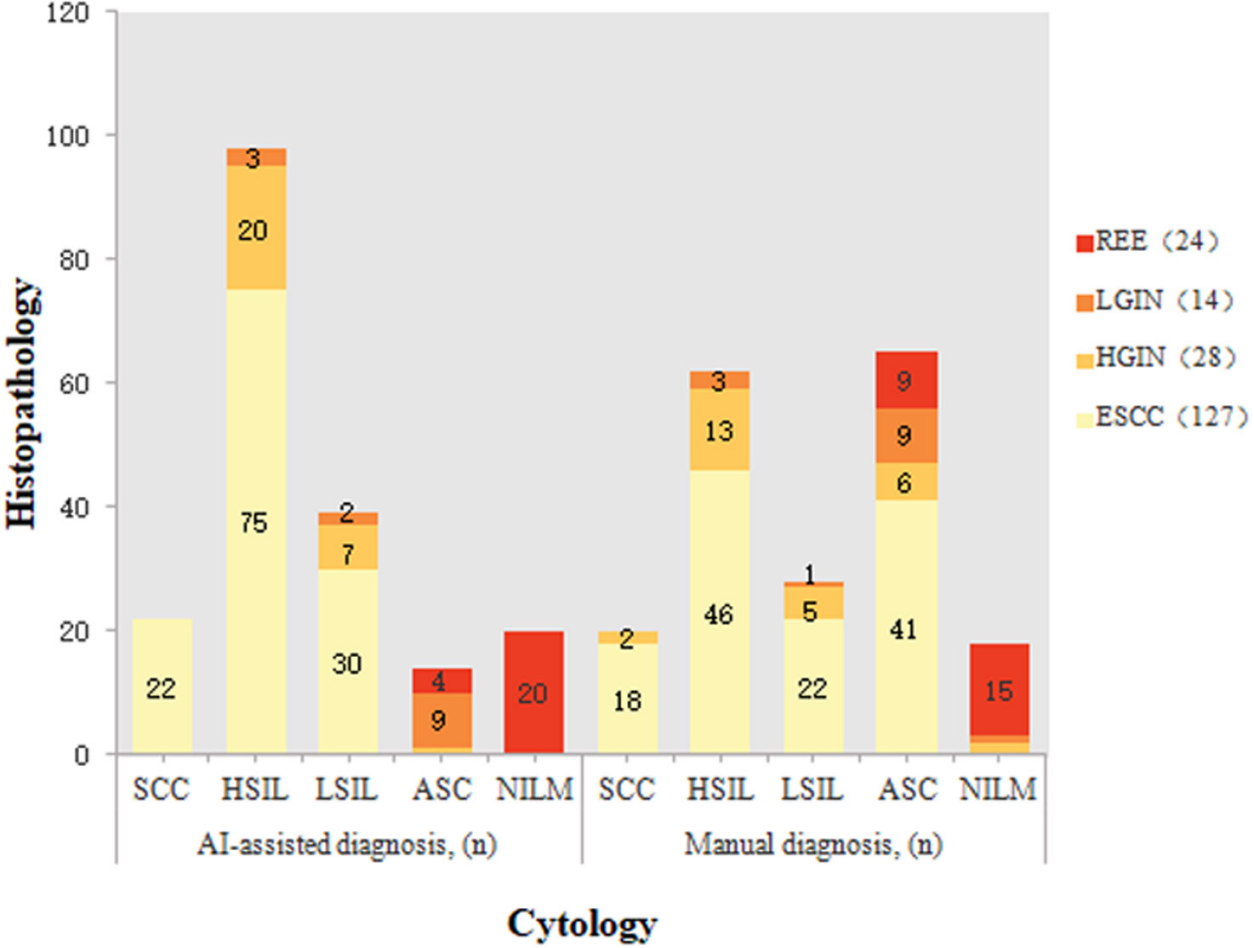
Correlation between histopathology and cytology. Results of AI‐assisted cytology and manual diagnosis were listed respectively. AI, artificial intelligence; SCC, squamous cell carcinoma; HSIL, high‐grade squamous intraepithelial lesion; LSIL, low‐grade squamous intraepithelial lesion; ASC, atypical squamous cell; NILM, non‐intraepithelial malignancy; ESCC, esophageal squamous cell carcinoma; HGIN, high‐grade intraepithelial neoplasia; LGIN, low‐grade intraepithelial neoplasia; REE, reflux erosive esophagitis.

As listed in Table 1 and Figure 3, 154 and 106 cases with early esophageal lesions were correctly identified by AI‐assisted diagnosis and manual diagnosis, respectively. For the interest of detecting early ESCC and HGIN (Table 2), AI‐assisted diagnosis demonstrated a significantly higher diagnostic accuracy (96.89% [95% CI, 92.38%–98.57%] vs 72.54% [95% CI, 65.85%–78.35%], *p* = 1.42 × 10^−14^), a higher sensitivity (99.35% [95% CI, 95.92%–99.97%] vs 68.39% [95% CI, 60.36%–75.48%], *p* = 7.11 × 10^−15^) and a higher NPV (97.06% [95% CI, 82.95%–99.85%] vs 40.96% [95% CI, 30.46%–52.31%], *p* = 1.42 × 10^−14^) than those of manual diagnosis. And, specificity (86.84% [95% CI, 71.12%–95.05%] vs 89.47% [95% CI, 74.26%–96.57%], *p* = 1.000) and PPV (96.86% [95% CI, 92.43%–98.84%] vs 96.36% [95% CI, 90.41%–98.83%], *p* = 1.000) were not significantly differed.

**TABLE 1:** Cytological detection for histopathologically confirmed esophageal lesions by AI‐assisted diagnosis and manual diagnosis

Histopathology (*n*)	Cytology									
	AI‐assisted diagnosis (*n*)					Manual diagnosis (*n*)				
	SCC	HSIL	LSIL	ASC	NILM	SCC	HSIL	LSIL	ASC	NILM
ESCC (127)	22	75	30	0	0	18	46	22	41	0
HGIN (28)	0	20	7	1	0	2	13	5	6	2
LGIN (14)	0	3	2	9	0	0	3	1	9	1
REE (24)	0	0	0	4	20	0	0	0	9	15

Abbreviations: ASC, atypical squamous cell; ESCC, esophageal squamous cell cancer; HGIN, high‐grade intraepithelial neoplasia; HSIL, high‐grade squamous intraepithelial lesion; LGIN, low‐grade intraepithelial neoplasia; LSIL, low‐ grade squamous intraepithelial lesion; NILM, non‐intraepithelial malignancy; REE, reflux erosive esophagitis; SCC, squamous cell cancer.

**TABLE 2:** Comparsion of diagnostic efficiency between AI‐assisted diagnosis and manual diagnosis

Diagnostic efficiency	AI‐assisted diagnosis	Manual diagnosis	*p* value
Accuracy (95%CI)	96.89% (92.38%–98.57%)	72.54% (65.85%–78.35%)	1.42 × 10^−14^
Sensitivity (95%CI)	99.35% (95.92%–99.97%)	68.39% (60.36%–75.48%)	7.11 × 10^−15^
Specificity (95%CI)	86.84% (71.12%–95.05%)	89.47% (74.26%–96.57%)	1.000
PPV (95%CI)	96.86% (92.43%–98.84%)	96.36% (90.41%–98.83%)	1.000
NPV (95%CI)	97.06% (82.95%–99.85%)	40.96% (30.46%–52.31%)	1.42 × 10^−14^

Abbreviations: NPV, negative predictive value; PPV, positive predictive value.

**FIGURE 4:**
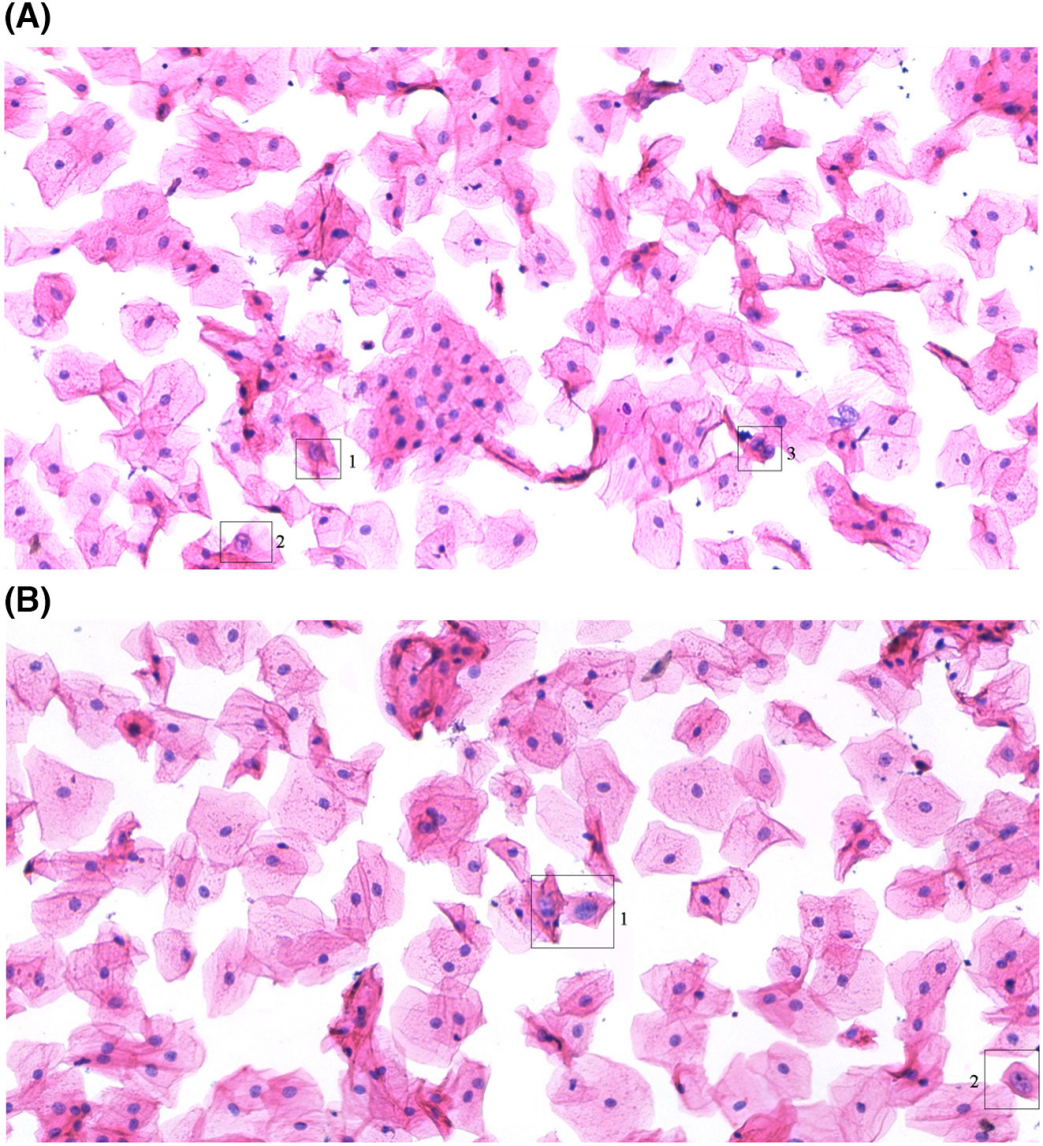
Samples of interpretive errors from manual diagnosis. (A) One case of underdiagnosed abnormal cells, which were shown in black boxes. 1,2: LSIL misdiagnosed as ASC, 3: HSIL misdiagnosed as ASC. (B) One case of misdiagnosis of abnormal cells. As shown in black box 1 and 2, HSIL was not identified by manual diagnosis. ASC, atypical squamous cell; HSIL, high‐grade squamous intraepithelial lesion; LSIL, low‐grade squamous intraepithelial lesion.

Slides from misdiagnosed cases, including one from AI‐assisted diagnosis and 49 from manual diagnosis, respectively, were re‐evaluated by three expert cytopathologists. The missed case by AI‐assisted diagnosis, which was HGIN instead of NLIM, was caused by inadequate staining and scanty abnormal cells. Whereas, 48 misdiagnosed cases by manual reading only were due to interpretive errors. Among these, 21 cases with LSIL‐or‐worse were underdiagnosed as ASC or NLIM due to lack of positive control cells for diagnosis. Twenty‐seven cases with LSIL‐or‐worse were missed due to few abnormal cells were present in all slides. By contrast, AI‐assisted reading altered the cytologists by presenting LSIL‐or‐worse abnormality. Typical samples of these cases are shown in Figure 4. The remaining one case was due to lack of sufficient numbers of abnormal cells.

### 3.3 Detection of HGIN and early ESCC in community‐based screening

According to cytological results, NILM, ASC, LSIL, HSIL, and SCC were present in 1845, 193, 57, 20, and 2 participants, respectively. The proportion of participants with abnormal cells by using LSIL‐or‐worse as diagnostic threshold was significantly lower than that by using ASC‐or‐worse (3.73%, [79/2117] vs12.84% [272/2117], *p* = 1.59 × 10^−58^). Based on endoscopic and histopathological outcomes, 1969, 88, 24, 23, and 13 participants were diagnosed as negative, REE, LGIN, HGIN and early ESCC, respectively. These results are shown in Table 3.

**TABLE 3:** Endoscopic and cytological outcomes of community‐based screening

Endoscopy and histopathology (*n*)	Cytology (*n*)				
	SCC (2)	HSIL (20)	LSIL (57)	ASC (193)	NLIM (1845)
ESCC (13)	2	6	4	0	1
HGIN (23)	0	14	6	1	2
LGIN (24)	0	0	18	5	1
REE (88)	0	0	20	61	7
Negative (1969)	0	0	9	126	1834

Abbreviations: ASC, atypical squamous cell; ESCC, esophageal squamous cell cancer; HGIN, high‐grade intraepithelial neoplasia; HSIL, high‐grade squamous intraepithelial lesion; LGIN, low‐grade intraepithelial neoplasia; LSIL, low‐ grade squamous intraepithelial lesion; NILM, non‐intraepithelial malignancy; REE, reflux erosive esophagitis; SCC, squamous cell cancer.

Changing the AI‐assisted diagnostic threshold from ASC‐or‐above‐level to LSIL‐or‐above‐level, the consistence between cytology and endoscopy was improved (40.51% [32/79] vs 12.13% [33/272], *p* = 1.54 × 10^−8^); the specificity (97.74% [95% CI, 96.98%–98.32%] vs 88.52% [95% CI, 87.05%–89.84%], *p* = 3.19 × 10^−58^) and PPV (40.51% [95%CI, 29.79%–52.15%] vs12.13% [95% CI, 8.61%–16.75%], *p* = 1.54 × 10^−8^) were also enhanced. Sensitivity (88.89% [95% CI, 73.00%–96.38%] vs 91.67% [95% CI, 76.41%–97.82%], *p* = 1.000) and NPV (99.80% [95% CI, 99.46%–99.94%] vs 99.84% [95% CI, 99.48%–99.96%], *p* = 1.000) were not significantly differed between these two settings (Table 4).

**TABLE 4:** Diagnostic performances in community‐based screening by using two settings of diagnostic threshold

Diagnostic performances	Diagnostic threshold		*p* value
	LSIL‐or‐worse	ASC‐or‐worse	
Consistence between cytology and endoscopy	40.51%	12.13%	1.54 × 10^−8^
Sensitivity (95% CI)	88.89% (73.00%–96.38%)	91.67% (76.41%–97.82%)	1.000
Specificity (95% CI)	97.74% (96.98%–98.32%)	88.52% (87.05%–89.84%)	3.19 × 10^−58^
PPV (95% CI)	40.51% (29.79%–52.15%)	12.13% (8.61%–16.75%)	1.54 × 10^−8^
NPV (95% CI)	99.80% (99.46%–99.94%)	99.84% (99.48%–99.96%)	1.000

Abbreviations: ASC, atypical squamous cell; LSIL, low‐grade squamous intraepithelial lesion; NPV, negative predictive value; PPV, positive predictive value.

The cost of upper gastrointestinal endoscopy including Lugol's iodine staining, histopathological diagnosis, and sponge‐based cytology were 600, 130, and 400 Yuan (RMB) per case, respectively. In this screening cohort, the per capita cost for detecting early ESCC and HGIN by using AI‐based cytology as pre‐endoscopy detection was significantly lower than that of endoscopy screening only (23682.7 ± 28.4, RMB Yuan vs. 35382.9 ± 19.0, RMB Yuan, *p* = 2.5843 × 10^−11^).

## 4 DISCUSSION

In this study, we firstly discussed the possibility and feasibility of LSIL‐or‐worse as the diagnostic threshold for cytological detection for histopathologically confirmed early ESCC and HGIN by using an AI‐assisted diagnosis platform. Also, its potential use as the primary screening method in a community‐based screening has been primarily addressed.

For many years, ASC has been applied as the diagnostic threshold in manual cytological diagnosis for detecting early esophageal squamous cell lesions.^6^, ^12^, ^15^, ^16^ Due to its low clinical significance, ASC is not a good diagnostic threshold for cytological detection for cancerous lesions,^4^, ^5^, ^17^ and many false‐positive cases were identified by using ASC as the threshold for cytological detection of abnormal cells. As a result, cytological detection and screening for ESCC were unsatisfactory by using this setting, by presenting a low diagnostic efficiency, poor sensitivity, specificity and PPV, and a low diagnostic accuracy.^6^ Although application of a rigid diagnostic threshold in cytological detection for cancerous lesions has yielded a more accurate outcome,^11^ data regarding this theme in early ESCC and HGIN is not available. In two recent previous studies,^12^, ^13^ we also adopted ASC‐or‐worse as the diagnostic threshold in AI‐assisted diagnosis, which was mainly due to some worries of misdiagnosis by using a higher one.

In this study, precise classification of esophageal epithelial squamous cells has been realized by using a well‐trained AI‐based automated recognition and classification. An accurate interpretation of cells makes it available that LSIL‐or‐worse could be set as the diagnostic threshold for detecting esophageal lesions. And, high efficiency and data homogeneity in cytological diagnosis have been achieved, as reflected by high diagnostic accuracy, high interobserver agreement, and a significantly shorter time effort for each slide. Compared to manual diagnosis only, the AI‐assisted diagnosis contributed to about 35% increase of diagnostic accuracy for histopathologically confirmed cases. Similar to a previous study,^11^ the misdiagnosis was also significantly decreased by AI‐based reading as auxiliary diagnosis. Thus, this AI‐assisted diagnosis is sufficient as a primary diagnosis for cytological detection for early ESCC and HGIN. Also, it makes cytological detection for such lesions a more accurate, convenient, and high‐efficient procedure. Therefore, we have provided a novel solution for a rigid diagnostic threshold in cytological detection of early ESCC and HGIN.

Currently, the outcomes of cytological screening for early ESCC and precursor lesions is poor, and its role as a primary screening before endoscopy is limited.^8^ One main reason is a low diagnostic accuracy and a low PPV.^6^, ^15^ And, immunohistochemistry staining^6^ or fluorescent in situ hybridization^14^ targeting p53 was performed as complementary examinations for the aim of increasing the real positive rate. Furthermore, the PPV of AI‐assisted cytological detection of early ESCC and HGIN in community‐based screening was about 12% by using ASC‐or‐worse for identifying potential positive cases.^12^, ^13^ By contrast, our data showed that a significantly smaller proportion of potential positive participants who need endoscopy examination for further confirmation were identified by using LSIL as the diagnostic threshold than that of ASC. Based on the final endoscopic and histopathological results, numbers of true positive cases with confirmed early cancerous lesions were not differed between two diagnostic thresholds. And, some false positive cases were excluded, and many unnecessary endoscopy examinations could be avoided. A higher PPV and a higher consistence between cytology and endoscopy were present. Also, specificity of our setting was superior to that by using ASC‐or‐worse. Adoption of a higher diagnostic threshold did not cause a reduction in sensitivity and NPV of cytological screening. Therefore, our study showed improved quality of cytological detection for early esophageal squamous lesions, which may be more suitable as a pre‐endoscopy screening for large‐scale screening for early esophageal squamous epithelial lesions.

The most interest of this study is that it makes large‐scale screening for early ESCC and its precursor lesions a more economical procedure. Because total incidence of ESCC is not high even in those areas with a high incidence rate, economic burden in population‐based screening for early ESCC and its precursor lesions should be considered. It has been reported that the positive rate of population‐based endoscopic screening is about 2% in those regions with an extremely high‐prevalence.^18^, ^19^ Due to the high cost of endoscopy and followed pathology, total and per capita cost for detecting early ESCC and HGIN in population‐based screening by endoscopy only is high‐costing. According to our result, average cost for detecting each positive case from large‐scale participants was significantly reduced about 30% by using this AI‐assisted diagnosis as a primary screening.

Although AI‐assisted cytology significantly reduced misdiagnosis by manual evaluation, one missed ESCC and two HGINs from community‐based screening were revisited. Endoscopic images revealed very small lesions (<5 mm in sizes), therefore, cytology was negative in these three cases. Subsequently, timely follow‐up cytology is recommended in participants with high risks of ESCC and negative cytological outcomes. Also, good quality control should be executed in sample collection and slides preparation in such procedures.^20^

A key limitation of this study is that the performance was carried out by one tertiary center. Possible bias in participant recruitment may be involved. Another limitation is that the community‐based screening was conducted in a region with a relatively low incidence of disease, as reflected by total positive rate of all identified early ESCC and HGIN at 0.71% (15/2117). However, the feasibility of this AI‐assisted diagnosis with a rigid diagnostic threshold has been verified owing to the high efficiency of a well‐trained AI‐based automated recognition and classification.

Taken together, we have developed an AI‐assisted diagnosis for automatically cytological detection of esophageal squamous lesions and validated its efficiency. A rigid diagnostic threshold has been firstly established in such detection. This automated classification also provides a novel pre‐endoscopy screening method for large‐scale screening of early ESCC and HGIN.

## AUTHOR CONTRIBUTIONS

Yadong Feng, Huazhong Shu, and Ruihua Shi conceptualized this study. Yadong Feng, Bin Yao, Kai Zhao, and Yan Liang performed cytology and endoscopy. Peilin Huang and Juncai Zang reviewed cytological and histopathological results and performed statistical analysis. Jie Song, Mengjie Li, and Xiaofen Wang were in charge of follow‐up and data collection. Yadong Feng drafted the original manuscript. Bin Yao, Kai Zhao, and Ruihua Shi edited the manuscript. All authors reviewed and approved the final edition of this manuscript.

## FUNDING INFORMATION

This work is funded by Jiangsu Provincial Special Program of Medical Science (BE 2019710 and BE 2018620), Nanjing Municipal Social Development Program (201803056), Medical Innovation Program of Jiangsu Commission of Health (2017XKJQW08), Major Project of Changzhou Municipal Commission of Health (ZD2019028) and Changzhou Municipal Social Development Program (CE20195001).

## CONFLICT OF INTEREST

The authors declare that there is no conflict of interest.Bin Yao andJuncai Zang are employees of Foreasy Tech Co. They are the main members of two projects of Jiangsu Provincial Special Program of Medical Science (BE 2019710 and BE 2018620). They do not have any financial arrangement for this research and the preparation of this manuscript.Bin Yao is pursuing a PhD degree at Southeast University.

## ETHICAL STATEMENT

The protocol conformed to the ethical guidelines of the 1975 Declaration of Helsinki, and has been approved by review board of Zhongda Hospital Southeast University, with Ref No. 2019ZDSYLL092‐P01. Written consent was obtained from each participant before enrollment into this study.

## Data Availability

The data that support the findings of this study are available from the corresponding author upon reasonable request.
